# A systematic analysis of a mi-RNA inter-pathway regulatory motif

**DOI:** 10.1186/2043-9113-3-20

**Published:** 2013-10-24

**Authors:** Stefano Di Carlo, Gianfranco Politano, Alessandro Savino, Alfredo Benso

**Affiliations:** 1 Department of Control and Computer Engineering, Politecnico di Torino, Torino, IT, Italy; 2 Consorzio Interuniversitario Nazionale per l’Informatica, Verres (AO), IT, Italy

**Keywords:** Regulatory networks, Network motifs, miRNA, Pathways

## Abstract

**Background:**

The continuing discovery of new types and functions of small non-coding RNAs is suggesting the presence of regulatory mechanisms far more complex than the ones currently used to study and design Gene Regulatory Networks. Just focusing on the roles of micro RNAs (miRNAs), they have been found to be part of several intra-pathway regulatory motifs. However, inter-pathway regulatory mechanisms have been often neglected and require further investigation.

**Results:**

In this paper we present the result of a systems biology study aimed at analyzing a high-level inter-pathway regulatory motif called Pathway Protection Loop, not previously described, in which miRNAs seem to play a crucial role in the successful behavior and activation of a pathway. Through the automatic analysis of a large set of public available databases, we found statistical evidence that this inter-pathway regulatory motif is very common in several classes of KEGG *Homo Sapiens* pathways and concurs in creating a complex regulatory network involving several pathways connected by this specific motif. The role of this motif seems also confirmed by a deeper review of other research activities on selected representative pathways.

**Conclusions:**

Although previous studies suggested transcriptional regulation mechanism at the pathway level such as the Pathway Protection Loop, a high-level analysis like the one proposed in this paper is still missing. The understanding of higher-level regulatory motifs could, as instance, lead to new approaches in the identification of therapeutic targets because it could unveil new and “indirect” paths to activate or silence a target pathway. However, a lot of work still needs to be done to better uncover this high-level inter-pathway regulation including enlarging the analysis to other small non-coding RNA molecules.

## Background

Systems biology is increasingly highlighting that a discrete biological function can only rarely be attributed to a single molecule. Instead, most biological characteristics arise from complex interactions among the cell’s numerous constituents, such as proteins, DNA, RNA and small molecules [1-3]. Understanding the structure and the dynamics of complex intercellular networks that contribute to the structure and function of a living cell is therefore mandatory.

The fast development of technologies to collect high-throughput biological data allows us to determine how different molecules interact with each other, leading to a proliferation of biological networks (e.g., protein-protein interaction, metabolic, signaling and transcription-regulatory networks). Several public and commercial network repositories including the WikiPathway database [4,5], the Ingenuity database [6], and the Kyoto Encyclopedia of Genes and Genomes (KEGG) [7,8], collect large amount of curated biological networks that can be explored and analyzed for high-level systemic analysis. None of these networks is independent, instead they form a complex network of networks that is responsible for the behavior of the cell. In this paper we concentrate on the key role that small non-coding RNAs, and in particular micro RNAs (miRNAs), have in this intricate biological network of networks.

Several results have been achieved in the past few years from the research of recurrent motifs in complex Gene Regulation Networks [9-18], highlighting the central role of miRNAs in governing specific regulation mechanisms at the network level [19-22]. In order to move toward the identification of higher-level mechanisms of transcriptional regulation, in this paper we performed a systemic analysis on a large set of well known biological networks to underline the presence of an inter-network regulatory motif in which miRNAs seem involved in a high-level regulatory activity among different networks. Rather then searching for pure topological motifs, available networks have been enriched with biological information from several public repositories to attempt to link obtained results to selected biological mechanisms [23].

**Figure 1:**
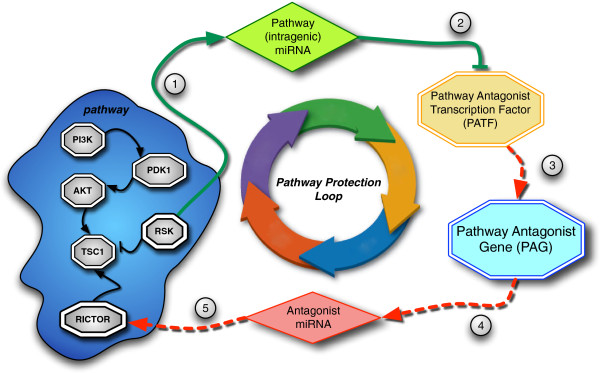
**Pathway Protection Loop.** Pathway Protection Loop (solid lines indicate active interactions; dashed lines indicate inhibited effects): 1) an activated pathway is host of one or more intragenic miRNAs that are therefore co-expressed with the pathway; 2) the pathway miRNAs target one or more PATF; 3) the down-regulated PATFs down-regulate the corresponding PAGs; 4) the down-regulated PAGs are not able to express the Antagonist miRNAs; 5) the down-regulation of the pathway genes by the Antagonist miRNAs is prevented.

Figure 1 shows the structure of the investigated network motif. According to this motif, the successful full activation of a regulatory network (pathway) not only depends on the correct expression of the pathway’s genes, but also on the expression of other genes potentially belonging to different networks that could interfere or dysregulate (down-regulate or silence) the pathway at some point. We call these genes the Pathway Antagonist Genes (PAGs). A possible way PAGs may interfere with the activity of a pathway is to express a set of miRNAs, in this context called Antagonist miRNAs, targeting and down-regulating some of the pathway’s genes. Interestingly, we discovered that, in several analyzed networks, the pathway intragenic miRNAs target and silence the transcription factors of the PAGs, thus creating a loop that seems designed to prevent PAGs from interfering with the pathway expression process. Given this characteristic we named this motif as Pathway Protection Loop (PPL).

When a PPL is present, its action is carried out in the following steps:

1. When the pathway is activated, one or more pathway genes co-express one or more intragenic miRNAs.

2. The intragenic miRNAs expressed by the pathway target one or more transcription factors of some of the PAGs. We call these transcription factors Pathway Antagonist Transcription Factors (PATFs). In some situations, the pathway intragenic miRNA may also target the pathway itself, but this mechanism belongs to well-studied intra-pathway regulations that are not the focus of this work.

3. The down-regulation of the PATFs has a repressive effect on the expression of the corresponding PAGs.

4. Down-regulated PAGs have lower ability to express the Antagonist miRNAs. It is worth here to remember that miRNAs have a post-transcriptional regulation role. Intragenic miRNAs that directly target the PAGs would not actually prevent the production of the related Antagonist miRNAs since miRNAs are expressed during transcription. The only way PPLs can form is therefore by mediating the PAGs down-regulation through their corresponding PATFs [24].

5. The reduced presence of Antagonist miRNAs contributes to the successful expression of the pathway genes, thus closing the protection loop.

An interesting characteristic of PPL is its hierarchical structure: a very small number of intragenic miRNAs (usually one or two) is able to “defend” the expression of either a large number or even the most important pathway genes.

This paper proposes an extensive systems biology study to analyze the existence and the characteristics of this new motif on a large set of public available networks. Results will show statistical evidence that this inter-network regulatory motif is very common in several classes of considered networks, and it can be used to identify an intricate set of links among networks thus building a high-level pathway to pathway interaction network. Finally, to further support the proposed research activity, literature mining allowed us to find clues of possible dysregulated PPLs in several papers targeting the study of tumors [25,26].

## Results and discussion

To assess the presence of PPLs we analyzed a set of networks for the *Homo Sapiens* species available in the KEGG database [8].

The KEGG database contains a set of 203 networks related to the *Homo Sapiens* species and represents one of the most curated and reliable source of pathway information. KEGG is unique for its focus and coverage of yeast, mouse, and human metabolic and signaling pathways [27]. All 203 pathways have been carefully analyzed in order to keep only representative and reliable networks. Human disease pathways have been excluded from the analysis since they represent deviations from correct behaviors that may change the mechanisms responsible for the formation of PPLs. Moreover, a set of few additional pathways not actually containing a regulatory network have been excluded, obtaining a final set of 158 pathways available for the analysis. All these 158 pathways have been manually checked and are all regulatory sub-networks. Each is, of course, part of THE regulatory network including the whole genome. Nevertheless, the separation in single “functional” pathways is necessary to make the problem manageable with the current tools.

The final set of considered KEGG pathways is reported in the two files Additional file 1 and Additional file 2 provided as additional files to this paper. The KEGG pathway repository contains several classes of networks describing a very large set of biological processes. The type of biological process, and consequently the involved actors (e.g., genes, proteins, metabolites, etc.) may bias the presence or the absence of PPLs. It has therefore been taken into account in our analysis. KEGG already categorizes all pathways according to a hierarchical ontology called the KEGG BRITE hierarchy. We exploited the first hierarchical level of this ontology to cluster all considered pathways into two main categories related to the ability of the corresponding nodes to be involved in miRNA mediated regulatory processes as will be discussed in the Statistical Analysis section. The first category contains 107 metabolic pathways while the second category contains 51 non-metabolic pathways (9 from KEGG cellular processes classification, 16 from KEGG environmental information processes classification, 6 from KEGG genetic information processes classification and 20 from KEGG organismal systems classification).

All pathways have been analyzed to search for the presence of PPLs resorting to a bioinformatics pipeline featuring the aggregation of information from several public on-line biological databases (see the Materials and methods section). To statistically analyze the existence of PPLs in the selected pathways we compared the obtained results with the ones gathered analyzing a population of randomly generated pathways. We generated a random population of 100 randomized networks. The size of each random network has been selected by first computing the mean (μ_size_) and the standard deviation (σ_size_) of the size of all networks in the KEGG dataset and by sampling a Normal distribution *N*(μ_size_, σ_size_) to obtain random network sizes comparable with the real ones. Genes composing each network have been then randomly selected from the Sanger Genecode database release 9 (Sanger) [28].

### Statistical analysis

We analyzed data obtained from our analysis using the R language and its environment for statistical computing [29]. The full set of analyzed data is available in the files Additional file 1 and Additional file 2 provided as additional files to this paper.

We first investigated if there is significant statistical difference in the frequency pathways manifest a PPL among the three considered groups of pathways (metabolic, non-metabolic, and random). Table 1 reports the contingency matrix indicating the frequency in which PPLs manifest in the three considered groups. A pathway is counted in the L column if it manifests at least one PPL, otherwise it is counted in the NL column. The number of PPLs observed in a pathway is not taken into account at this stage but it will be analyzed later in this section. Since miRNAs play a pivotal role in the formation of a PPL, the way miRNA targets are selected must be carefully taken into account. As better explained in the Materials and methods section, we search for potential miRNA targets resorting to microRNA.org, which performs this operation applying a computational approach providing a score (mirSVR score) to measure the reliability of each prediction. In order to reduce false positives at minimum, we restricted our target search to the mirSVR predictions labeled as “Good mirSVR score, Conserved miRNA” and “Good mirSVR score, Non-conserved miRNA” that represent the most reliable predictions available into microRNA.org. Moreover, we further restricted miRNA targets only considering high-score predictions (mirSVR < -0.3, see Materials and methods section), taking into consideration the aggregated data provided in the Additional file 1.

**Table 1:** Statistical analysis of PPL occurrence in pathways (mirSVR < -0.3): PPL occurrence contingency matrix

**Group of pathways**	**Presence of loops**		**Total**
	**L**	**NL**	
#Metabolic	10 (9.3), [15.8]	97 (90.7), [49.7]	107
#Non-metabolic	28 (54.9), [44.4]	23 (45.1), [11.8]	51
#Random	25 (25), [39.8]	75 (75), [38.5]	100
Total	63	195	

Rows indicate the three groups of pathways while columns indicate pathways manifesting PPLs (L) and pathways where PPLs have not been found (NL). Frequencies have been calculated considering miRNA targets with mirSVR < -0.3.

() = percentage in rows; [] = percentage in columns.

In order to find relationships among groups of pathways, frequencies reported in Table 1 have been analyzed with pairwise Pearson’s Chi-squared test using the R chisq.test command. Furthermore, Holms adjustment of the obtained p-values [30] has been carried out with the p.adjust procedure (see Table 2).

**Table 2:** Pairwise Pearson’s Chi-square tests among all possible pairs of pathway groups (i.e., non-metabolic vs. random, metabolic vs. non-metabolic and metabolic vs. random) for PPLs identified with mirSVR < -0.3. p-values have been adjusted applying Holms adjustment

	**Metabolic**	**Non-metabolic**
Non-metabolic	χ^2^ = 36.7867	
	p = 3.953649 × 10^-09^	
Random	χ^2^ = 7.9363	χ^2^ = 11.9768
	p = 4.845243 × 10^-03^	p = 1.077333 × 10^-03^

Pearson’s Chi-squared test among the three groups points out that there is significant statistical dependence between rows and columns of the contingency matrix reported in Table 1 (*χ*^2^ = 38.8678, d.f. 2, p = 3.631× 10^-09^), thus confirming our hypothesis that PPLs manifest with different frequencies based on the considered groups. To better understand where differences among groups lie, post-hoc analysis has been performed. We performed a chi-squared test considering all possible pairs of groups (i.e., non-metabolic vs. random, metabolic vs. non-metabolic and metabolic vs. random). Analyzing the obtained results reported in Table 2, we noticed that pathways including PPLs appear with a significant higher frequency in non-metabolic pathways (55%) than in metabolic pathways (9%) (*χ*^2^ = 36.7867, p = 3.953649 × 10^-09^). This insight is in accordance with the study done in [31], that suggests the presence of a “universe” of miRNAs deeply involved in the regulation of signaling pathways, which represent a large portion of the non-metabolic pathways group. Overall, considering KEGG signaling pathways only, about 71% of them contain PPLs. Instead, as expected, metabolic pathways exhibit a reduced percentage of PPLs due to the high presence of metabolites in their nodes, which are unable to express pathway intragenic miRNAs that are responsible for the creation of PPLs. Pathways including PPLs also appear with significant higher frequency in non-metabolic pathways (55%) than in random pathways (25%) (*χ*^2^ = 11.9768, p = 1.077333 × 10^-03^) confirming our hypothesis that the establishment of this motif is not due to chance. Moreover, the frequency of pathways with PPLs is higher in the random group compared to the metabolic group (*χ*^2^ = 7.9363, p = 4.845243 × 10^-03^). Again, this is non surprising at all. As already stated, metabolic pathways are in large part formed by metabolites unable to express the intragenic miRNAs required to create a PPL. Differently, random pathways include nodes which are randomly sampled from the full set of genes available in the Sanger Genecode database and have a higher probability to include genes expressing miRNAs potentially able to establish a PPL.

To further analyze the characteristics of the proposed motif, we also investigated if we can observe statistical difference in the number of PPLs per pathway among the different groups (this information is available in the Additional file 1). We analyzed the distribution of this variable for Normality with the Kolmogorov-Smirnov test using the R lillie.test procedure. The result confirmed the lack of normality (D = 0.4335, p < 2.2 × 10^-16^). Eventually, the Kruskal-Wallis test, a non-parametric analysis of variance [32], has been performed resorting to the R kruskal.test procedure.

Kruskal-Wallis rank sum test underlines that there is statistical difference in the number of loops among the three groups of pathways (H = 37.6374, d.f. 2, p = 6.716 × 10^-09^). Again to better understand the differences among the different groups we performed post-hoc analysis running a set of Mann–Whitney U tests among pairs of different groups applying Holms p-value adjustment resorting to the R pairwise.wilcox.test procedure. Results of this analysis, reported in Table 3 once more, confirm that non-metabolic pathways manifest a higher number of PPLs compared to both metabolic (p = 1.0 × 10^-09^) and random (p = 0.0028) pathways. The same way, metabolic pathways manifest a lower number of loops compared to random pathways (p = 0.0028) further confirming the previous analysis on frequency of networks manifesting PPLs.

**Table 3:** **Mann–Whitney *U *** **test on the PPL numerosity among all possible pairs of pathway groups (i.e., non-metabolic vs. random, metabolic vs. non-metabolic and metabolic vs. random) for PPLs identified with mirSVR < -0.3. p-values have been adjusted applying Holms adjustment**

	**Metabolic**	**Non-metabolic**
Non-metabolic	p = 1.0 × 10^-09^	
Random	p = 0.0028	p = 0.0028

As previously mentioned, it is clear that the reliability of our findings may also depend on the reliability of the miRNA target predictions, since the more targets are considered the more loops may appear. Although in our analysis we already restricted the set of considered targets in microRNA.org and we further filtered targets selecting only those with mirSVR < -0.3, to better understand this bias, we repeated the overall PPL identification and the related statistical comparison with randomized networks considering an increased cut-off value for miRNA target prediction scores (mirSVR < -0.6) thus reducing the number of predicted targets. Aggregated data for this mirSVR threshold are available in the Additional file 2 and the related statistical analysis is reported in Tables 4, 5 and 6. The statistical analysis performed on these aggregated data confirms the results obtained with the mirSVR < -0.3 threshold.

**Table 4:** Statistical analysis of PPL occurrence in pathways (mirSVR < -0.6): PPL occurrence contingency matrix

**Group of pathways**	**Presence of loops**		**Total**
	**L**	**NL**	
#Metabolic	7 (6.5), [14.0]	100 (93.5), [48.08]	107
#Non-metabolic	24 (47.05), [48.0]	27 (52.95), [12.98]	51
#Random	19 (19), [38.00]	81 (81), [38.94]	100
Total	50	208	

Rows indicate the three groups of pathways while columns indicate pathways manifesting PPLs (L) and pathways where PPLs have not been found (NL). Frequencies have been calculated considering miRNA targets with mirSVR < -0.6.

() = percentage in rows; [] = percentage in columns.

**Table 5:** Pairwise Pearson’s Chi-square tests among all possible pairs of pathway groups (i.e., non-metabolic vs. random, metabolic vs. non-metabolic and metabolic vs. random) for PPLs identified with mirSVR < -0.6. p-values have been adjusted applying Holms adjustment

	**Metabolic**	**Non-metabolic**
Non-metabolic	*χ*^2^ = 33.4281	
	p = 2.2218396 × 10^-08^	
Random	χ^2^ = 6.2143	χ^2^ = 11.7142
	p = 1.26723 × 10^-02^	p = 1.240482 × 10^-03^

**Table 6:** **Mann–Whitney *U *** **test on the PPL numerosity among all possible pairs of pathway groups (i.e., non-metabolic vs. random, metabolic vs. non-metabolic and metabolic vs. random) for PPLs identified with mirSVR < -0.6. p-values have been adjusted applying Holms adjustment**

	**Metabolic**	**Non-metabolic**
Non-metabolic	p = 9.5 × 10^-09^	
Random	p = 0.0049	p = 0.0029

Pearson’s Chi-squared test on the contingency matrix reported in Table 4, that indicates the frequency in which PPLs manifest in the three considered groups of pathways with this new mirSVR threshold, still confirms that there is significant statistical dependence between rows and columns (χ^2^ = 36.3039, d.f. 2, p = 1.308 × 10^-08^), thus confirming that even reducing the set of miRNA targets to the ones with higher score we still observe that PPLs manifest with different frequencies based on the considered groups. Table 5 further confirms this result when post-hoc analysis is performed to analyze differences among pairs of groups. Finally, Kruskal-Wallis rank sum test on the number of loops among the three groups of pathways confirms statistical differences also in this case (H = 34.1145, d.f. 2, p = 3.91 × 10^-08^), and this difference is confirmed also in Table 6 when Mann–Whitney U tests are used for post-hoc analysis among the different pairs of groups.

This outcome is particularly interesting since it highlights that the identified PPLs mainly involve high-score miRNA gene predictions, thus adding reliability to our findings.

### Interaction among networks

**Figure 2:**
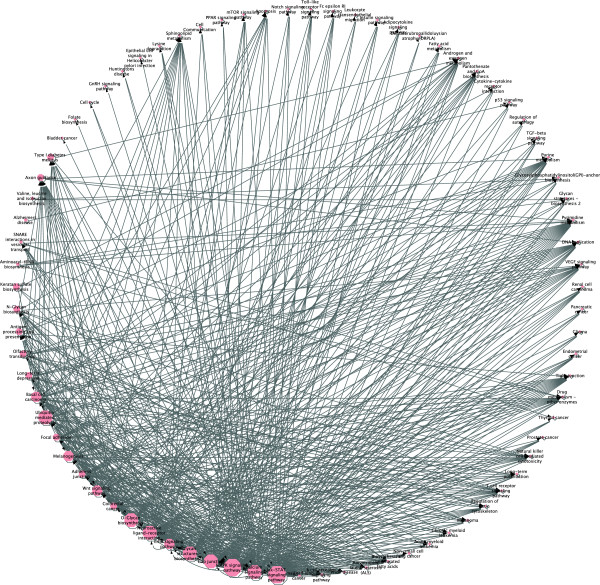
**Network of pathways linked by PPLs.** Network of pathways highlighting the intricate regulation mechanism introduced by PPLs.

After performing statistical analysis on the presence of PPLs in this section we analyze how PPLs actually constitute a miRNA based inter-network regulation mechanism that can be used to build a complex network of networks that involves several pathways. Starting from the full list of identified PPLs that is available at http://www.testgroup.polito.it/index.php/component/k2/item/184-ppl-list we built the network reported in Figure 2 that is also available as a Cytoscape 2.8 session file in the Additional file 3.

Each node of the network represents a KEGG pathway. Two types of nodes are available: (1) hexagonal nodes represent pathways in which PPLs have been detected, (2) rhomboidal nodes are pathways in which no PPLs have been detected but containing at least one PAG. A directed weighted edge connects two pathways if a PPL generated from the first pathway targets a PAG contained in the second pathway. The weight of the edges represents the number of PPLs connecting the two pathways. Furthermore, each node is labeled with an additional parameter reporting the number of PAGs of the pathway that have not been detected in any of the KEGG pathways.

The network reported in Figure 2 clearly shows how the PPL motif creates a very intricate regulatory mechanism among different pathways. 79 pathways are involved in this mechanism and 552 edges identify interactions between pathways involving at least a PPL.

By analyzing the nodes generating PPLs using the Cytoscape network analyzer plugin, it is also possible to highlight that on average, each pathway generating PPLs is connected to 25.111 pathways thus confirming the complexity of the identified motif, which involves the cooperation of several pathways.

This enforces the idea that miRNAs cover different, and often even conflicting, roles in gene regulatory networks. From this perspective, we can identify three important and complementary roles for miRNAs in gene regulation: the first is the well-known intra-pathway regulatory role targeting genes belonging to the pathway itself; the second is an inter-pathway down-regulatory effect, where miRNAs expressed by a pathway directly silence mRNAs from genes belonging to pathways that may be biochemically or functionally incompatible with the pathway that is being expressed; the third is an indirect up-regulatory function where miRNAs, thanks to the PPL motif, indirectly contribute to the pathway up-regulation by down-regulating the Transcription Factors of its PAGs.

In the remaining of this paper two pathways manifesting the PPL motif will be analyzed in detail. The full list of pathways where PPLs have been identified has been provided as additional material to this submission in the form of dot graph files.

### PPLs in mTOR signaling pathway

**Figure 3:**
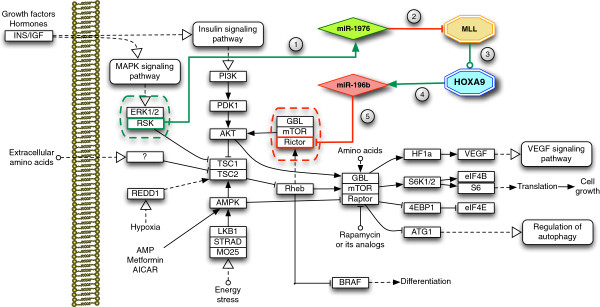
**mTOR signaling pathway (#hsa04150).** TOR signaling pathway (#hsa04150) including the identified PPL (3/31/09 release). (1) The pathway host-gene (RSK) expresses a protective intragenic miRNA (miR-1976) which (2) acts against the expression of the MLL transcription factor. (3) MLL is responsible for HOXA9 transcription, that (4) leads to the expression of miR-196b which, if expressed, (5) would target RICTOR, dysregulating the mTORC2 complex in which RICTOR plays a central role.

The simplicity of the PPL protection mechanism can be appreciated in Figure 3, which shows the PPL detected in the KEGG mTOR signaling pathway (#hsa04150 - http://www.genome.jp/kegg-bin/show_pathway?hsa04150).

The mTOR signaling pathway has been identified as a hub. It integrates the output of several upstream pathways, including insulin, growth factors and amino acids [33], as well as cellular nutrition, energy levels and redox status [34]. The mTOR pathway is actually under the analysis of several research units. Its pharmacological targeting looks like an effective method for acting against multiple types of cancer (e.g., leukemia, glioblastoma, myelodysplasia breast, hepatic and pancreatic [35,36]), in which the mTOR pathway appears dysregulated [37]. The mTOR pathway contains two main complexes: mTORC1 and mTORC2. mTORC1 has been largely analyzed, whereas mTORC2 (regulated by insulin, growth factors, serum, and nutrient levels [38]) has been less clearly investigated. In order to better understand the role of the mTORC2 complex several knockouts experiments have been performed on its genes and direct interactors. In particular, the RICTOR gene has been highlighted as responsible for metastasis and inhibition of growth factors [39]. Its down-regulation is directly linked to the reduced phosphorylation of AKT and PKC, which leads to an impaired differentiation of Th2 cells, producing IL-4, IL-5, IL-10, and IL-13, responsible for strong antibody production, eosinophil activation, and inhibition of several macrophage functions, providing phagocyte-independent protective responses [40]. Dysregulated type 1/type 2 cytokine production and their skewed development have been implicated in the progression of multiple immune disorders including asthma [41,42], leukemia [43], and other cancers [44]. This also leads to a renewed interest in using type 1 and type 2 cytokines as markers of human immune function.

Interestingly, the RICTOR gene appears as an actor in the PPL identified within the mTOR pathway. As shown in Figure 3, the PPL is composed of a pathway host-gene (RSK) expressing a protective intragenic miRNA (miR-1976) which acts against the expression of the MLL transcription factor. MLL is responsible for HOXA9 (one of the PAGs) transcription, leading to the expression of miR-196b (the Antagonist miRNA), which would target RICTOR, if expressed, dysregulating the mTORC2 complex. This result seems to strongly confirm the central role of MLL, the HOXA cluster (HOXA9), and both miR-196b and miR-1976 in Acute Lymphoblastic Leukemia (ALL), as presented by Schotte et al. [25,45]. The PPL overall suggests that aberrant miR-196b expression may in fact contribute to leukemogenesis, as dysregulation of HOX genes were shown to directly induce leukemia in mice [46]. The results presented by Schotte et al. [25,45] may suggest that many of the observed dysregulations are compatible with disruption of the observed PPL. The common assumption is that miRNAs discovered in a pathological context have a dysregulatory role; in this case the PPL suggests instead that miR-1976 [25] may have a protective role, and its slight over expression may indicate the mTOR pathway attempt to protect itself.

The most common pathological rearrangements of MLL (t(4;11), t(11;19), t(9;11) and t(1;11)) may mislead the proper miR-1976 regulatory function because the MLL translocation may imply changes in its miRNAs binding sites. Popovic et al. [47] showed that leukemogenic MLL fusion proteins cause over- expression of miR-196b, while treatment of MLL-AF9 transformed bone marrow cells with miR-196 specific antagomir abrogates their replating potential in methylcellulose. This may suggest that miR-196b function is necessary for MLL fusion-mediated immortalization and it may justify the fact that the mTOR pathway protects itself by not allowing its expression through the PPL. Similarly, the same work shows that the level of miR-196b is decreased up to 14-fold in the absence of MLL, thus confirming the down-regulatory role of miR-1976 on MLL.

To further validate these observations, we analyzed the expected level of expression of interactors in ALL disease retrieved from Gene Expression Atlas (http://www.ebi.ac.uk/gxa/). The expression of both RSK and RICTOR appears compatible with the identified PPL: (1) RSK (E-MTAB-62 experiment, filtered by ALL) shows an upper regulation in ALL (p = 0.002), and it may confirm the attempt of the pathway to protect its correct behavior maximizing the production of protective miR-1976 by over-expressing its host gene; (2) RICTOR (E-MTAB-37 experiment, filtered by ALL) appears globally down (p = 9.07e - 4), accordingly with the observed miR-196b up-regulation.

The reliability of our findings depends on the reliability of the miRNA target predictions, since the more targets are considered, the more loops may appear. In order to consider only reliable predictions we filtered miRNA targets for mirSVR score lower than -0.3 (see Materials and methods section). Relaxing this threshold may identify additional PPLs with weaker target affinity. In this case, we identified three additional low-score miR-1976 targets in the mTOR pathway, which are PAG Transcription Factors involved in PPLs. However, their role and possible involvement in the disease dynamics are still under investigation.

### PPLs in the antigen processing and presentation (APP) pathway

**Figure 4:**
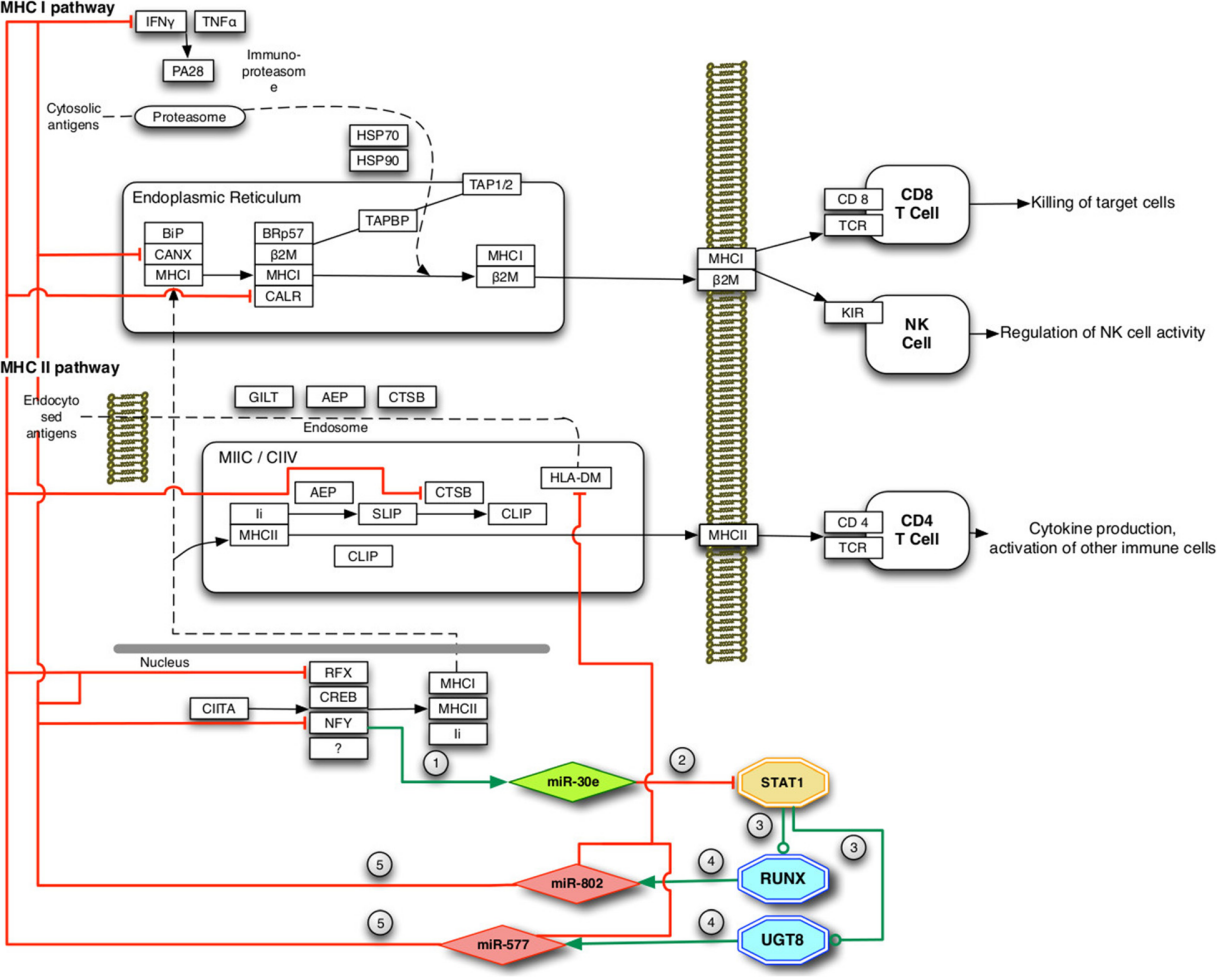
**Antigen processing and presentation (APP) pathway (#hsa04612).** Antigen processing and presentation (APP) pathway (#hsa04612) including the identified PPLs. The pathway host-gene (NFY-C) expresses a protective intragenic miRNA (miR-30e6) which (2) acts against the expression of the STAT1 transcription factor. (3) STAT1 is responsible for RUNX and UGT8 transcription, that (4) leads to the expression of miR-802 and miR-577 which, if expressed, (5) would target IFN-?, CANX, CALR, CTSB, RFX, HLA-DM, NFY-C.

Another very interesting example of pathway including PPLs can be found in the antigen processing and presentation (APP) KEGG pathway (#hsa04612-http://www.genome.jp/kegg-bin/show_pathway?hsa04612) reported in Figure 4 that shows several PPLs. Identified PPLs are originated by NFY-C that co-expresses the intragenic miRNA miR-30e which targets the STAT1 transcription factor. STAT1 is responsible for the transcription of two PAGs (1) RUNX and (2) UGT8, that respectively co-express miR-802 and miR-577, responsible for the loop closure.

APP is composed of two inner pathways responsible for synthesis of major histocompatibility complexes I and II (MHCI and MHCII), which are responsible for cell destruction (when MHCI expression is low) and specific immunization (MCHII).

The NFY complex is well known for peptide presentation in antigen presenting cells [48] and for being highly enriched in specific phases of the cell-cycle [49], thus playing a central role in cell control and maturation. The identified PPLs look compatible with the NFY behavior observed in mammals, in which the complex acts as an on/off switch by post-transcriptional mechanisms, and other more subtle post-translational regulations [50].

miR-30e, located in the intronic region of NFY-C and co-transcribed with its host gene, has been highlighted as responsible for maintaining differentiated cell phenotypes. For instance, the knock out of miR-30 miRNA family induces epithelial-mesenchymal transition of pancreatic islet cells [51]. Moreover, miR-30e is under-expressed in breast, head, neck, and lung tumors, with experimental evidences confirming that its ectopic expression suppresses uncontrolled cell growth [52]. This regulatory role seems compatible with the PPL behavior in which miR-30e is the only miRNA deputed to protect the pathway. Thus, the miR-30e dysregulation may lead to a wrong antigen exposition, which does not allow proper T cells to target the dysregulated cells, avoiding apoptosis driven either by CD8+ or NK T cells [53].

miR-30e directly targets the STAT1 transcription factor that belongs to the signal transducers and activators of transcription family. STAT1 is involved in up-regulation of genes (interferon stimulated genes) in response to different interferon based stimulation. In particular, after IFN-γ stimulation, STAT1 forms homodimers or heterodimers with STAT3 for binding with GAS (interferon-gamma activated sequence) promoter elements and their further regulation [54]. This feedback loop targeting IFN-γ, may suggest a fine tuning loop between IFN-γ and STAT1.

STAT1 promotes two PAGs: RUNX1 and UGT8. RUNX1, also known as AML1 or CBFA2, is a transcription factor that regulates the fate of hematopoietic stem cell populations and is generally regulated by 2 enhancers, which are tissue specific and drive the binding of lymphoid or erythroid regulatory proteins [55]. RUNX1 takes part in cell fate process mediating the transition of an endothelial cell into a haematopoietic cell. Evidences in RUNX1 knock out mice showed that primitive erythrocytes displayed a defective morphology, and the size of blast cell population was substantially reduced [56]. At least 39 forms of RUNX1 mutations are implicated in various myeloid malignancies. Chromosomal translocations involving RUNX1 are associated with several types of leukemia including AML [57]. As for MLL in the mTOR pathway previously discussed (see sub-section PPLs in mTOR signaling pathway), single nucleotide polymorphism (SNP), chimerism and translocation may invalidate the standard PPL regulation machinery, causing unexpected misbehaviors.

UGT8 encodes for an enzyme involved in glycosphingolipids synthesis, in particular galactosylceramides (GalCer lipids), which are involved in a variety of cellular processes including differentiation, cell-cell interaction, and transmembrane signaling [58,59]. It is also noticeable that UGT8 is mainly localized in the endoplasmic reticulum, but not in the Golgi complex, nor in the plasma membrane [60]. The same characteristic applies to the final PPL target, the HLA-DM complex targeted via miR-577, which is also only localized in the endoplasmic reticulum.

Furthermore, previous studies highlighted that an induced dose-dependent inhibition of GalCer expression on the cell surface, after treatment with recombinant gamma-interferon (rIFN-γ), caused reduced viral (HIV- 1) infection by decreasing GalCer synthesis and expression [61]. This may be explained by a certain level of competition between IFN-γ and UGT8, in accordance with the identified PPL. As for RUNX1, also UGT8 is known to have multiple non-synonymous SNPs which could affect structures and/or biological functions of the respective gene products [62].

miR-802, co-expressed with RUNX, targets multiple pathway genes: IFN-γ, NFY-C, CANX, and the HLA-DM complex. It is worth noticing that among its targets we find NFY-C, which is responsible for the PPLs initiation. CANX is a chaperone protein responsible for protein folding and quality control. It retains unfolded or mis-folded proteins in the endoplasmic reticulum, in order to have only well assembled proteins in the cytoplasm. CANX also controls the folding of the MHC class I alpha chain. This central role in the MHCI synthesis makes it a possible critical target in PPL dysregulation. The HLA-DM protein, another chaperone, finally, is targeted by both miR-802 and miR-577. HLA-DM regulates the peptides that bind to MHCII, and controls/presents the antigen in antigen presenting cells. It plays a central role in the MHCII complex stability by favoring more stable peptide-MHC complexes. Dysregulation of HLA-DM is associated with negative prognosis in breast cancer, since patients with tumors that co-express HLA-DR, Ii and HLA-DM have improved recurrence-free survival as compared with patients with tumors that express HLA-DR and Ii in the absence of HLA-DM [26], and, accordingly to the discussion of miR-30e, HLA-DM negative patients show a general paucity of infiltrating CD3+, CD4+ and CD8+ T cells [63]. Under expression of HLA-DM is also proven in autoimmune processes in Rheumatoid Arthritis [26] and Hodgkin Lymphoma [63].

## Conclusions

The discovery of the Pathway Protection Loops is suggesting a level of transcriptional regulation at the pathway level not fully investigated before. Studies conducted on specific miRNAs such as the one published by Barik [64] confirm the presence of this type of regulatory motif, but a high-level analysis such as the one proposed in this paper is still missing.

The understanding of this and other higher-level regulatory motifs could, for example, lead to new approaches in the identification of therapeutic targets because it could unveil new and “indirect” paths to activate or silence a target pathway.

A lot of work still needs to be done to better uncover this high-level inter-pathway regulation. miRNA are not the only small RNAs that are involved in regulatory mechanisms. For example, ceRNA have been recently identified as miRNA down-regulators [65]. Unfortunately data available on these new mechanisms is still very limited and therefore it is not yet possible to include genome-wide investigations like the one presented in this paper.

## Materials and methods

To study the characteristics and properties of PPLs, we designed a software pipeline that, combining pathway data available via PathwayAPI [66] with the Micronome data extracted from public databanks (e.g., Microrna.org [67], miRBase [68], etc.), is able to search for miRNA mediated interactions at the pathway level, thus searching for the existence of PPLs.

The full software pipeline that is available at (http://www.testgroup.polito.it/index.php/bio-menu-tools/item/185-pathway-rotection-loops-finder) has been implemented as a collection of PHP classes, given the need of interfacing our software with several web based sources of information. All collected data have been saved into a unified relational database used for mining information about PPLs.

### Pathway data sources

The search for the existence of the PPL motif starts from the analysis of a collection of pathways. Several public and commercial pathway resources currently exist on the web. However, these biological databases are very diverse, making it extremely laborious to carry out even simple queries across databases [69]. To overcome with this limitation, pathway related information have been retrieved through Pathway API [70]. Pathway API is an aggregated database combining and unifying databases from three major sources of information: (1) the WikiPathway database [5], the (2) Ingenuity database [6] and the (3) KEGG [8]. One of the main advantages of Pathway API is the normalization of the network nodes that are all consistently translated and named using the corresponding NCBI Gene ID [70], thus enabling an easy data integration with the other data sources considered in this work.

### Micronome and gene interaction data sources integration

**Figure 5:**
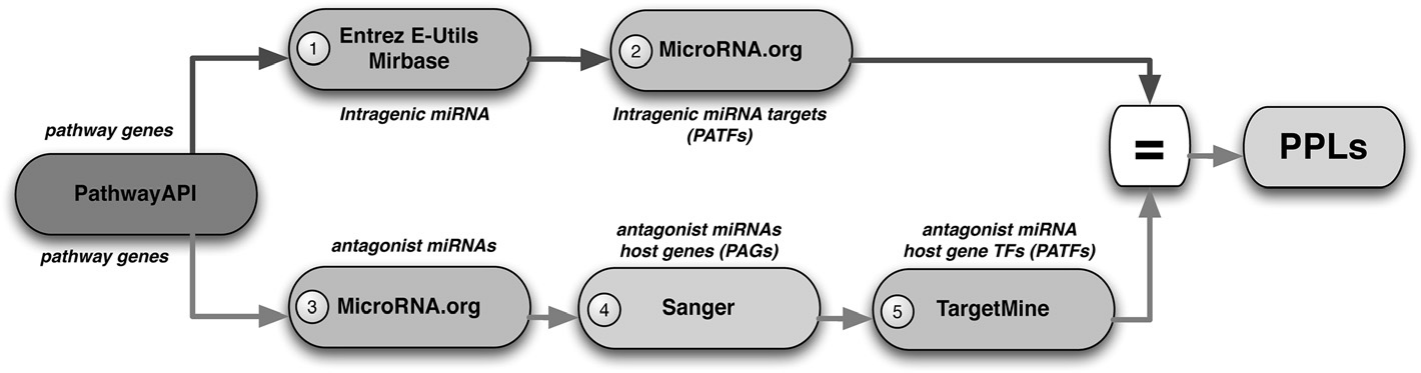
**PPL Identification PIPELINE.** Computational pipeline for the identification of pathway protection loops.

Figure 5 highlights the data sources and computational steps performed in our pipeline for the identification of PPLs in a pathway. The pipeline basically consists of two information retrieval flows. The two flows are applied to each gene composing the target pathway. The outcomes of these two flows are then intersected to identify the presence of PPLs.

Several external data sources are employed in the proposed pipeline, namely:

1. the Entrez E-Utils web API (e-Utils) [71],

2. the miRBase database release 18 (miRBase) [66]

3. the microRNA.org database (microRNA.org) [67,72],

4. the TargetMine database [73],

5. the Transcription Factor encyclopedia (TFe) [74], and

6. the Sanger Genecode database release 9 (Sanger) [28].

In several cases, these databases use different convention for identifying specific entities (e.g., genes). Whenever possible, information from the cited databases have been dumped into a local unified relational database and all entries have been then preprocessed to unify the different identifiers. Working with a local dump of the information also allowed us to speed-up the information retrieval process which requires a massive access to these information sources.

### Intragenic miRNA identification

To identify miRNAs co-expressed with the pathway genes we restricted our search to the set of intragenic miRNA. Intragenic miRNA represent around 50% of the mammalian miRNAs [75-79]. Most of these intragenic miRNA are located within introns of protein coding genes (miRNA host genes) and are referred to as intronic miRNA, whereas the remaining miRNAs are overlapping with exons of their host genes and are thus called exonic miRNA. Moreover the majority of intragenic miRNAs are sense strand located while only a very small portion is anti-sense strand located. Our analysis considers intronic and exonic miRNAs both sense and anti-sense strand located.

We assume that intragenic miRNA are in general co-expressed with their related host-genes as supported by previous studies [75,80-83]. Recently Chunjiang et al. [84] also suggested that evolutionary conserved intragenic miRNA tend to be co-expressed with their host genes more likely than poorly conserved ones. This consideration could further refine the outcome of our analysis, however at the current stage it has not yet been implemented in our pipeline.

Intragenic miRNAs are retrieved through the miRBase database. miRBase is a searchable database of published miRNA sequences and annotations. About 94.5% of the available mature miRNA sequences considered in this paper have experimental evidence, thus representing a reliable source of information. Each miRNA entry in miRBase is correlated with the related information on the genetic location that is exploited to identify the host genes.

To identify intragenic miRNA of a given host gene we first search for the coordinate of the gene using the e-Utils. Once obtained the gene coordinates we search for all miRNAs with coordinates embedded in the ones of the gene resorting to miRBase.

### Intragenic miRNA targets identification

We searched for potential targets of each identified intragenic miRNA resorting to microRNA.org. microRNA.org searches for miRNA targets applying the miRanda algorithm [85]. The miRanda algorithm identifies potential binding sites by looking for high-complementarity regions on the 3′UTRs. The scoring matrix used by the algorithm is built so that complementary bases at the 5′ end of the miRNA are rewarded more than those at the 3′ end. The resulting binding sites are then evaluated thermodynamically, using the Vienna RNA folding package [86] and each prediction is finally associated with a down-regulation score named mirSVR score [87]. Newer miRanda versions [88] implement a strict model for the binding sites that requires almost-perfect complementarity in the seed region with only a single wobble pairing, thus increasing the prediction accuracy. Other miRNA target databases such as TargetScan [89] use different prediction algorithms that aim at filtering many false positives from the beginning of the prediction process. However, the availability of the mirSVR score in microRNA.org provided us an additional degree of freedom to investigate the robustness of our prediction when changing the way microRNA targets are filtered. The second advantage offered by microRNA.org compared to other repositories such as TargetScan is the possibility of downloading the full database in a relational form. Given the amount of queries required by the proposed analysis this was a mandatory requirement to keep the computation time into a reasonable range.

To work with reliable predictions and limit the amount of returned miRNA-gene interactions, during the analysis we restricted our search to the microRNA.org “Good mirSVR score, Conserved miRNA” and “Good mirSVR score, Non-conserved miRNA” with negative mirSVR score lower than -0.3/-0.6. Given the selected intragenic miRNA name, searching for the targets simply requires an SQL query into the microRNA.org database.

### Antagonist miRNA identification

Antagonist miRNAs are miRNAs that target one of the genes of the pathway and similarly to the Intragenic miRNA targets can be retrieved through microRNA.org. Given the NCBI GeneID we query the microRNA.org database to identify the set of miRNAs targeting the gene. Query to microRNA.org at this step follows the same filtering rules on the mirSVR score applied for the identification of the intragenic miRNA targets.

### Antagonist miRNA host gene identification

The identification of an antagonist miRNA host gene follows an inverted flow compared to the one employed to identify the pathway intragenic miRNAs. For each antagonist miRNA we identify the related coordinates using miRBase, and, given the coordinates, we search into Sanger for a gene whose coordinates embrace the one of the considered miRNA. Genes identified at this step represent potential PAGs.

### Antagonist miRNA host gene TF identification

As already mentioned in the introduction of this paper, miRNAs have a post-transcriptional regulation role [90]. Intragenic miRNAs that directly target the PAGs would not actually prevent the production of the related Antagonist miRNAs since miRNAs are expressed during transcription whereas the down-regulatory action is post-transcriptional. However, the expression of miRNAs can be activated or repressed by transcription factors of the related host genes, which therefore can serve as upstream regulators of miRNA [24]. For each antagonist miRNA host gene we therefore search for the related transcription factors. Searching for the transcription factors of the antagonist miRNA host genes is a critical step due to the limited availability of information from public databases that may strongly reduce our ability of identifying PPLs. For this reason we tried to integrate more than one data source in our search using two databases: (1) TargetMine and (2) TFe.

Both TargetMine and TFe provide web services to access the related database. To speed up the analysis all information contained in these two repositories have been downloaded and merged into a single database table containing relations between TFs and related target genes.

To download the information contained in TargetMine, we retrieved from Sanger the full list of NCBI GeneIDs considered in our analysis. For each geneID we then searched for TF targeting the selected gene through the REST service http://targetmine.nibio.go.jp:8080/targetmine/service/template/results?name=Gene_TFSource&constraint1=Gene&op1=LOOKUP&format=xml&&extra1=H.+sapiens&value1=⟨targetgeneid⟩. The resulting xml formatted information has then been processed and integrated in the database.

A similar approach has been applied to download the information provided by TFe. The list of all TFs available in TFe has been downloaded through the REST service http://www.cisreg.ca/cgi-bin/tfe/api.pl?code=all-tfids. For each TF in the list, the list of targets has been computed calling the REST service http://www.cisreg.ca/cgi-bin/tfe/api.pl?code=entrez-gene-id&tfid=⟨TFID⟩. The resulted information has ben finally added to the local database and joined with the ones provided by TargetMine.

With the availability of a local database, searching for TFs targeting a given host gene simply requires to query the related database tables.

## Competing interests

The authors declare that they have no competing financial interests or other conflicts of interest.

## Authors’ contributions

Alfredo Benso and Alessandro Savino coordinated the overall study and participated in the result validation. Stefano Di Carlo mainly worked on the statistical validation and Gianfranco Politano carried out the PPL analysis on the two selected pathways. All authors contributed equally to this work. All authors read and approved the final manuscript.

## Declarations

This work has been partially supported by Grant No. CUP B15G13000010006 awarded by the Regione Valle d’Aosta for the project: “Open Health Care Network Analysis” and by the Italian Ministry of Education, University & Research (MIUR) (Project PRIN 2010, MIND).

## Supplementary Material

Additional file 1 **File containing aggregated data about the number of loops identified in the considered pathways.** Analysis is performed with mirSVR < -0.3 threshold on the miRNA target identification. Click here for file

Additional file 2 **File containing aggregated data about the number of loops identified in the considered pathways.** Analysis is performed with mirSVR < -0.6 threshold on the miRNA target identification. Click here for file

Additional file 3 **Cytoscape session file containing the network of interaction among pathways connected by PPLs.** It requires Cytoscape v.2.8 to be displayed. Click here for file
